# Improving patient flow of people with rheumatoid arthritis has the potential to simultaneously improve health outcomes and reduce direct costs

**DOI:** 10.1186/s12891-016-1362-7

**Published:** 2017-01-07

**Authors:** R. Puchner, R. Hochreiter, H. Pieringer, A. Vavrovsky

**Affiliations:** 1 Rheumatologist in Private Practice, Wels, Austria; 2 Institute for Statistics and Mathematics, Vienna University of Economics and Business Administration, Vienna, Austria; 3 2nd Department of Medicine, General Hospital Linz and Faculty of Medicine, Johannes Kepler University Linz, Linz, Austria; 4 Academy for Value in Health, Vienna, Austria

**Keywords:** Cost of illness, Delivery of care, Rheumatoid arthritis, Health care research

## Abstract

**Background:**

In our current economic climate of scarce resources there is a lot of debate around the best - and most efficient - way of delivering care, which points patients towards the right physician at the earliest opportunity.

The aim of the study was to assess whether an improvement in the interdisciplinary management of rheumatoid arthritis (RA) has the potential to simultaneously improve health outcomes and reduce costs.

**Methods:**

In a first step, we modelled the ways which lead patients with RA to the correct diagnosis, and the relevant specialist, respectively. On average, a patient experiences 3 GP visits before referral to a specialist. We compared this situation against a reconfiguration of current practice towards a more proactive identification and referral method with initiation of care by a rheumatologist early in the disease. We evaluated the impact of this reconfiguration on the number of RA patients diagnosed and the costs associated with the diagnostic process.

**Result:**

Using data on epidemiology and Austrian practice patterns, we estimate a total of 5294 people with undifferentiated arthritis per year, of which 1765 suffer from RA. Modelling for diagnostic accuracy, we found that 1200 of these patients are initially misdiagnosed in a primary care setting and 95 at a rheumatologist. Our model found that a reconfiguration of current practice towards an approach of more integrated care has the potential to be not only cost-effective, but cost-saving: EUR 100,188 could be saved annually by exclusively adopting the new approach.

**Conclusions:**

Our results show that by better directing the flow of people with RA, simultaneous clinical and economic benefits may be reaped:.

## Background

Inflammatory rheumatic diseases occur frequently across all age groups, job categories and social classes. They cause frequent sick leave and occupational disability [1, 2]. Diseases such as rheumatoid arthritis (RA) are characterized by their chronic and progressive nature and may lead to premature loss of joint function. Joint damage can occur early in the disease: after 2 years about 75% of patients have already developed joint damage with erosions [3, 4]. The need for early diagnosis and prompt therapeutic measures is evident and beyond any controversy nowadays, and an integral part of diagnostic paths and therapeutic guidelines [4, 5].

In the last decade, due to a better understanding of the pathogenesis of rheumatoid arthritis and its progression, highly effective drugs have revolutionised treatment. Until the end of the 1990s a reduction in the number of swollen joints and a reduction of pain intensity was an accessible and acceptable goal. Today, rheumatologists aim for remission and a symptom free status respectively [4, 6].

In our current economic climate of scarce resources, it has become mandatory to eliminate any potential for duplication of diagnostic efforts. This has led to a lot of debate around the best - and most efficient - way of delivering care, which points patients towards the right physician at the earliest opportunity. While this is the order of the day for care in general, it is especially important for people with inflammatory arthritis. Joint destruction may happen early and progress rapidly.

Insufficient patient management at the primary care level may lead to delayed diagnosis due to delayed referral to and therapy by a specialist and, subsequently, joint destruction [1, 3, 5].

Moreover, RA is not a straightforward disease to diagnose due to its nature and epidemiology. Comparing the number of RA patients in Austria and their general practitioner (GP) counterparts makes it evident that an Austrian primary care physician is confronted with 0.4 incident patients with RA per year (that is, dividing the number of office-based GPs by the number of incident RA patients in Austria). This number is in line with published German studies [7, 8]. It is well established that diagnostic accuracy is a function of frequency - and for diseases as infrequent as RA a variety of approaches exist that try to stimulate more rapid and integrated cooperation between GPs and specialists [9–11].

All of these approaches promote a close interaction between GPs and specialists to deepen the understanding of RA and to establish easily applicable referral criteria in suspected cases of RA.

The purpose of the present study was to assess whether an improvement in the interdisciplinary patient management of RA has the potential to simultaneously improve health outcomes and reduce costs. Early identification and referral of RA patients to a rheumatologist and rapid initiation of appropriate therapy is associated with a better disease outcome. There is unequivocal evidence that early treatment with the goal of achieving remission has the potential to reduce impairments in work productivity and daily activities [4, 12]. However, to receive treatment, patients have to get in touch with the right specialist first. Any retardation at this point in the care continuum may lead to delayed initiation of DMARD therapy resulting in radiographic joint damage which would have been preventable [13]. Van der Linden et al showed that RA patients, who were assessed within 12 weeks from symptom onset, were associated with less joint destruction and a higher chance for remission compared to patients with longer waiting times. [4].

Furthermore, diagnostic redundancies and physician visits will be reduced which may lead to lower overall costs.

## Methods

Previously the British National Audit Office published a report on two health economic models investigating the impact of earlier diagnosis and treatment of RA patients in England [14]. The effects evaluated included direct costs, employment and the quality of life. We adopted this approach for the present study to investigate whether an improved referral pattern of suspected incident RA patients would be associated with better patient care as well a reduced economic burden for the health care system.

Taking into account country-specific differences in patient management, one of the aforementioned English models served as the starting point of our analysis.

### Patterns and probabilities of practice and patient flow

In a first and fundamental step, we modelled the different ways, which lead patients with RA to the correct diagnosis, and the relevant specialist, respectively. We reconstructed the patient flow up to diagnosis in Austria, from onset of symptoms, presentation to GP or rheumatologist, GP referral to specialists, until, ultimately, RA diagnosis.

**Fig. 1:**
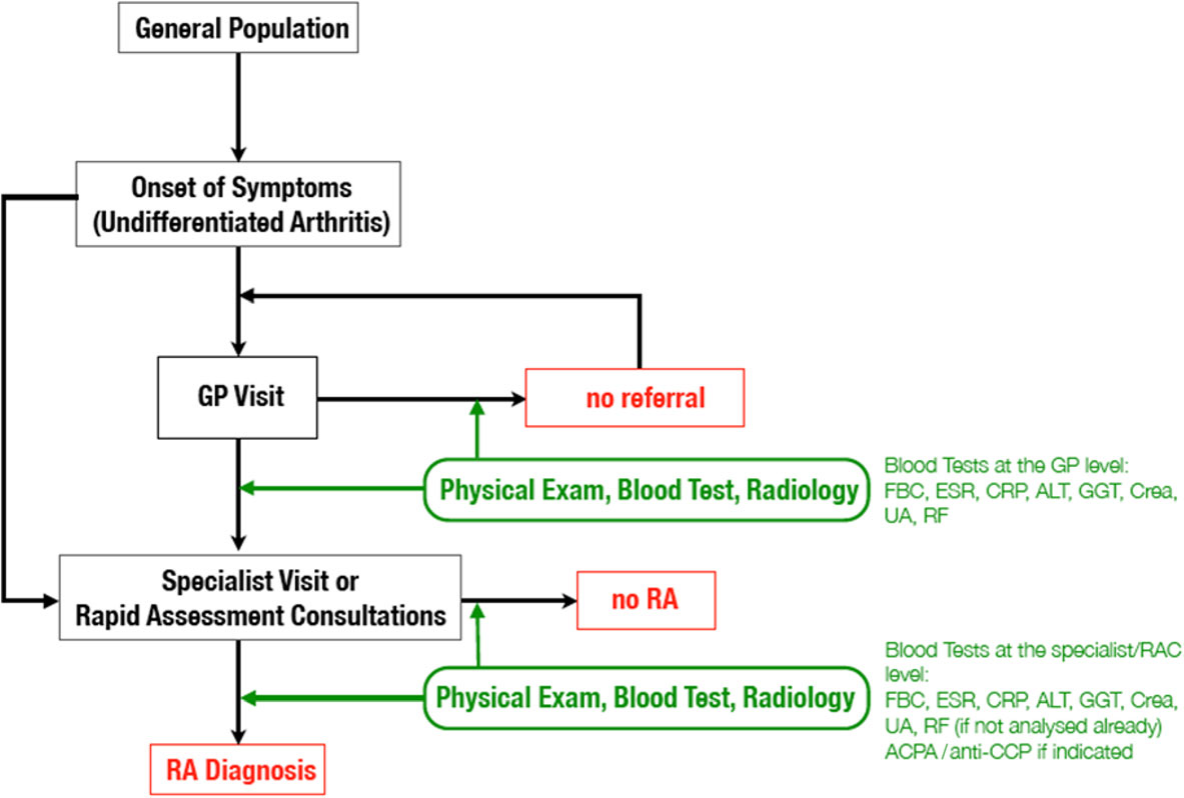
Current patient pathway in Austria. Abbreviations: ESR, erythrocyte sedimentation rate; CRP, C-reactive protein; FBC, full (=complete) blood count; ALT, alanine transaminase; GGT, gamma-glutamyl transferase; UA, uric acid; Crea, creatinine; RF, rheumatoid factor; ACPA, antibodies against citrullinated protein/peptide antigens

Figure 1 depicts the different steps and measures taken at the respective contact points. Expert interviews with both GPs and rheumatologists served as the basis for the decision tree [9].

After onset of symptoms, a patient has the option to consult with his/her GP, to see a specialist, i.e. a rheumatologist, directly, and to take advantage of the existing hospital-based “Immediate Access Rheumatology Clinics” [10] or office-based “Rapid Assessment Consultations (RAC)” [11] (to date only in Vienna and Upper Austria; not yet established nationwide). Data on practice patterns and diagnostic steps taken at the different points of care were taken from three published studies [10, 11, 15] and verified with practicing rheumatologists [9].

In the Austrian health care system, it is certainly intended that patients experiencing new symptoms of a disease see their GP first, however, there is no formal gate-keeping mechanism in place and patients are free to see both an office-based as well as a specialist in a clinic right away.

A GP who sees a patient with symptoms of RA is faced with two courses of action: immediate referral to a rheumatologist or watchful waiting, which most likely includes a blood analysis, the prescription of painkillers and a follow-up appointment. Previously we could show [15] that, on average, RA patients visit their GP 3 times before referral. This, in turn, could affect not only costs associated with diagnosis, but also radiographic progression.

To compare the current situation to a new approach, we first modelled the number of people with RA, diagnosed within 3 months of symptom onset, under current practice [15] as well as the costs associated. We then compared this situation against a reorganization, which will be summarised as the rapid assessment consultation, or RAC approach. The RAC aims at promoting fast diagnosis of early RA by fostering rapid identification and referral. This service includes an initial examination carried out by an experienced (office-based) rheumatologist. Regardless of the duration of symptoms, every patient is seen within 1 week (with or without referral), if an inflammatory rheumatic disease is suspected or the patient/referrer stresses the urgent need for a visit. Both a tentative diagnosis and a proposal for further medical care were given to the patients. In the case of a genuine rheumatologic disorder, immediate therapy was initiated. This approach has already been evaluated in Vienna and with a cooperative of office-based rheumatologists in upper Austria, who established an immediate access network offering brief initial assessments for patients with musculoskeletal problems [10, 11]. A description of the current or “traditional” patient flow is depicted in Fig. 1.

We evaluated the impact of this change on the number of RA patients diagnosed within a certain timeframe as well as on the costs associated with diagnosis. Table 1 shows the assumptions used in the model.

**Table 1:** Patterns and probabilities of practice and patient flow used in the model

Patterns of Practice and Patient Flow		
Probability of seeing GP first after onset of symptoms	0.8	[15]
Number of GP visits in case of no diagnosis or no referral	3	[15]
Probabilities of Patient Flow		
Probability of seeing GP first after onset of symptoms	0.8	[15]
Probability of seeing specialist first after onset of symptoms^a^	0.13	[15]

^a^7% consulted an orthopedic surgeon or an internist first (= 0.07)

### Epidemiology

The incidence and prevalence of RA varies across populations and disease definitions. In North America and Northern Europe, the annual incidence rate was estimated at 20–50 cases per 100,000 population and the prevalence at 0.5–1%. [16]. In 1994, the annual incidence was 36 per 100,000 in women and 14 per 100,000 in men; the prevalence was 0.8% of the adult population in the UK [17].

Table 2 summarises the demographic data [http://www.statistik.at/web_de/statistiken/index.html] and incidence rate estimates used in the model.

**Table 2:** Epidemiology data used in the model

Epidemiology		Reference
Austria’s mid-year male population, male, as per July 15, 2013	3,181,396	[http://www.statistik.at/web_de/statistiken/index.html].
RA: yearly incidence per 100,000 males	14	[17].
Austria’s mid-year female population, female, as per July 15, 2013	3,442,605	[http://www.statistik.at/web_de/statistiken/index.html].
RA: yearly incidence per 100,000 females	36	[17].
Probability of person presenting with symptoms of undifferentiated arthritis (UA)	0.00186	[14]

As there is no data on the epidemiology of RA in Austria, we also used data from Wiles et al from 1999, who estimate the yearly incidence of RA to be 24.5/100,000 for men and 54/100,000 for women, as the basis for the second scenario we modelled [18].

### Statistical methods

To compare the current situation in Austria with other scenarios, i.e. different parameterisations, we set up a probabilistic simulation model. Parameters are on the one hand probabilities concerning the disease within the population as well as approximations of various costs on the other hand, e.g. cost of treatment (both GP and specialist). An important parameter is the time a patient needs until he is receiving appropriate treatment. To this end the time to treatment is divided into five brackets, i.e. a certain probability whether a patient receives treatment within 3 months, 6 months, 12 months, 24 months, and above respectively is specified.

We assumed that patients are always in one of a finite number of discrete health states, and all events are transitions from one state to another. Transitioning between the health states is measured by the probabilities described in detail above. Moreover, we included resource use at each state. For each scenario, the total cost of the diagnostic process is derived by multiplying volumes times the unit cost of service provision.

The calculations and simulations have been conducted using the statistical computing package R [19].

## Results

### Probability values of inflammatory arthritis, of diagnosis after symptom onset by point in time and of correct diagnosis by physician group

Probability values used in modelling were obtained from literature reviews and expert opinion. Table 3 summarises the parameters used as well as their sources [10, 15].

**Table 3:** Probability of Inflammatory Arthritis, of diagnosis after symptom onset by time point, of correct diagnosis by physician group

	*P*	Reference
Probability of Inflammatory Arthritis		
Probability of a patient presenting with IA (RA or other IA)	0.6438	[10]
Diagnostic Probabilities		
Probability of RA diagnosis within 3 months	0.28	[15]
Probability of RA diagnosis within 4 to 6 months	0.16	[15].
Probability of RA diagnosis within 7 to 12 months	0.14	[15].
Probability of RA diagnosis within 13 to 24 months	0.17	[15]
Probabilities of Diagnostic Skill		
Probability of correct diagnosis by GP	0.15	[15]
Probability of correct diagnosis by specialist	0.73	[15]
Probability of correct diagnosis by specialist	0.76	[10]

Our model includes all people who may have undifferentiated arthritis (UA) (defined as people presenting with symptoms similar to inflammatory arthritis which could be inflammatory, but also non-inflammatory arthritis; studies suggest that the incidence of UA is three times the incidence of RA) [14, 20]. To fully capture the effects of a closer cooperation between GPs and specialists, we had to incorporate unintended effects on people with non-inflammatory arthritis with similar symptoms presenting to rheumatologists. We included the financial impact of both inflammatory and non-inflammatory patient groups and assessed diagnostic skill using data from a study by Gärtner et al [10].

We designed a one-way sensitivity analysis considering various relevant variables, especially the relationship between visits to a GP and the RAC approach, the average amount of GP visits as well as the incident rate, i.e. the relationship between inflammatory arthritis (IA) and UA within the population. It allowed for an integration of the pricing of false-positives and also supported a deeper general understanding of all parameters. Various scenarios have been deployed to gain insight into cost trends. Based on our base scenario and given a uniform distribution of patients to either GP or the RAC approach, an increase of average visits to the GP from 3 to 5 increased the total costs by EUR 41,745. Changing the current practice towards early referral of each and every patient with suspected RA to a specialist has the potential to be truly cost-saving, i.e. costs of EUR 51,041 can be saved.

### Costing

Our analysis takes the perspective of a third party payer in Austria, the Federation of Austrian Social Insurance Institutions (*Hauptverband der Sozialversicherungsträger*).

We compared the costs to the health system which are incurred at each stage of the diagnostic process. The following costs to the Federation of Austrian Social Insurance Institutions are included in the model: GP consultations; consultations by specialists, laboratory tests, both those carried out by GPs prior to referral and those carried out by specialists after referral (antibodies against citrullinated protein/peptide antigens (ACPA), C-reactive protein (CRP), tests for rheumatoid factor (RF), erythrocyte sedimentation rate (ESR), complete blood count (CBC), alanine transaminase gamma (ALT), glutamyltransferase (GGT), uric acid (UA), creatinine (Crea)). No treatment or monitoring costs after diagnosis are included in the present analysis.

Due to the perspective chosen for our analysis, indirect costs such as loss of productivity and earnings due to inability to work were not considered.

We adhered to the local national guidelines for health economic analyses and therefore used the weighted average of tariffs of four Austrian sickness funds [21]. Table 4 details the costs used in the model and their sources.

**Table 4:** Costs used in the model

Costs of Physician Visits	Weighted Tariff
GP visit	€27.83
Specialist visit	€31.15
Costs of Diagnostic Procedures	
Rheumatoid factor (RF)	€3.66
C-reactive protein (CRP)	€3.70
Erythrocyte sedimentation rate (ESR)	€1.84
Antibodies against citrullinated protein/peptide antigens (ACPA)	€14.33
Complete blood count (CBC)	€4.08
Alanine Transaminase gamma (ALT)	€2.22
Glutamyltransferase (GGT)	€2.64
Uric acid (UA)	€2.67
Creatinine (Crea)	€2.58

**Fig. 2:**
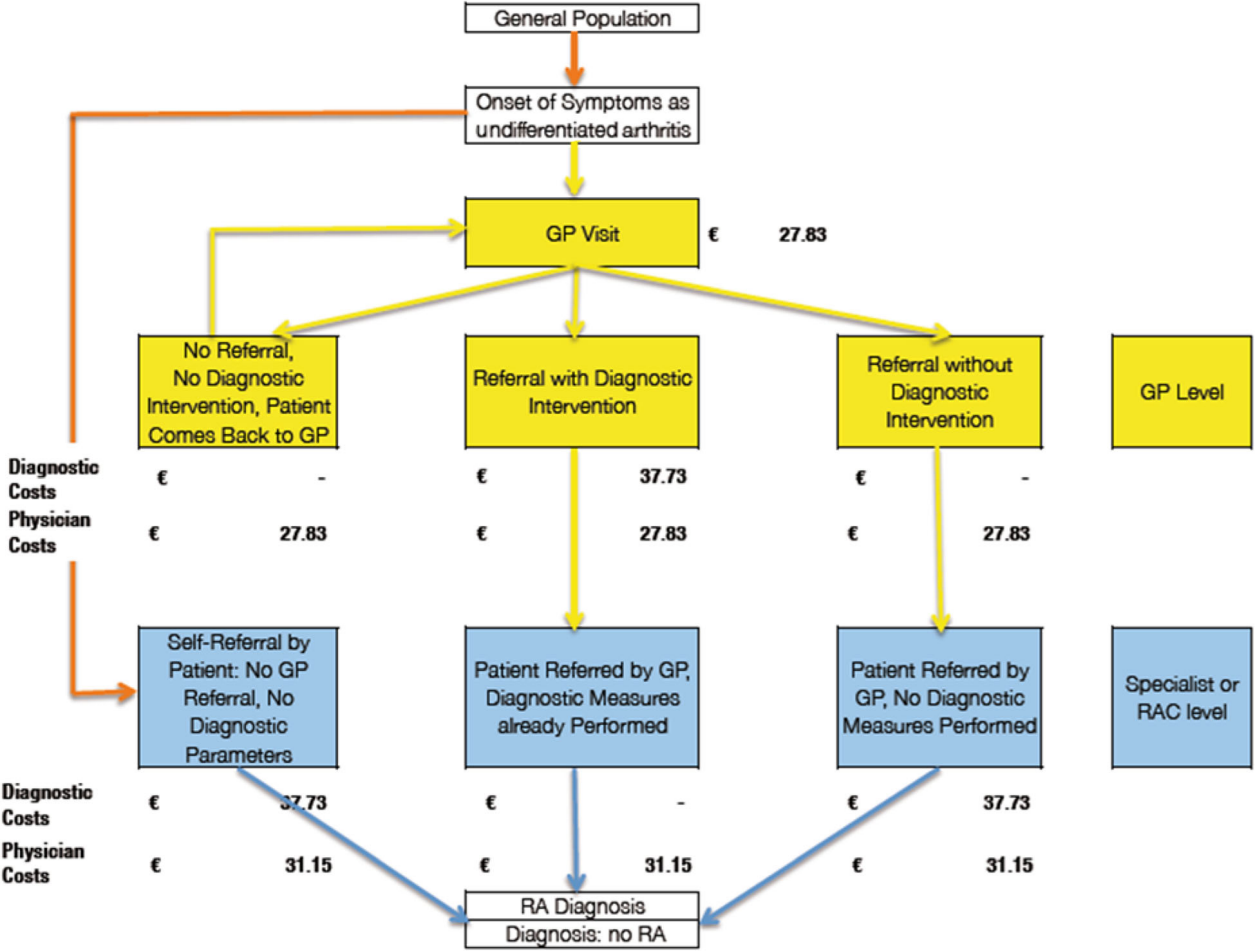
Costs incurred at each step of the patient pathway. Yellow boxes indicate GP activity, blue boxes indicate specialist activity. Abbreviations: RAC, rapid assessment consultation

The cost items included refer to the diagnostic parameters worked out by an expert panel of primary care physicians and rheumatologists [10]. Figure 2 depicts the steps of the patient flow used in the model as well as the associated costs of each step. Yellow boxes indicate activities taken at the GP level, while blue boxes contain information on what happens at the specialist level.

Combining the parameters listed in Tables 2 and 3, we estimate a total of 5294 people with undifferentiated arthritis in Austria, of which 1765 suffer from RA.

Modelling for diagnostic accuracy, we found that 1200 of these patients are currently misdiagnosed in a primary care setting. Inflammatory arthropathies are not straightforward to diagnose - even when consulting a specialist, it is expected that 95 RA patients are not correctly diagnosed. However, there is a marked increase of diagnostic accuracy at the specialist level (see Table 5).

**Table 5:** Results of our health economic modelling of a reconfiguration of patient flow of Austrian patients with suspected RA

	Patients (*n*)
All undifferentiated arthropathies	5,294
All RA (male and female)	1,765
Not (yet) diagnosed with RA by primary care physicians	1,200
Not (yet) diagnosed with RA by rheumatologists	95
Cost impact of existing referral pattern with suggested modified approach	EUR
Cost savings GP visits	100,188

These annual savings accrue as the sum of reduced GP visits and reduced diagnostic analyses taken at the GP level

In summary, our model shows that by adopting an RAC approach and changing current practice towards early referral of each and every patient with suspected RA to a specialist has the potential to be not only cost-effective, but cost-saving: EUR 100,188 could be saved annually, by exclusively adopting the new integrated approach. These savings accrue from a combination of reduced GP visits and reduced diagnostic analyses taken at the GP level.

Adopting the data on epidemiology suggested by Wiles et al [18] mentioned above, we found that the number of incident RA patients in Austria would rise to 2765 and henceforth cost-savings of EUR 156,961 under the new approach.

## Discussion

By increasing diagnostic accuracy, patients will receive disease-modifying anti-rheumatic drug (DMARD) treatment earlier, which offers the potential to halt radiographic damage. Joint destruction is in turn responsible for patients’ inability to work and function, both in their professional and private lives [13, 22, 23].

Huscher et al [24] showed that annual costs differ by Health Assessment Questionnaire (HAQ) level – poor functional status leads to higher direct costs. This proves the notion that disease activity does indeed impact costs. Radner et al [12] in their recent analysis found that the same holds true for indirect costs treated at a tertiary care centre in Austria.

We found that restructuring care for patients with suspected RA by providing primary care physicians with clear referral guidelines, a list of diagnostic measures which can be performed before the first appointment with a specialist [9] and specific contact points (eg Early Arthritis Clinics) is less expensive and improves care at the same time.

In health economics, for any intervention to be considered cost-saving, the approach under consideration must, at the same time, be less expensive and improve clinical parameters. This is a rare occurrence: Cohen et al [25] found that fewer than 1 in 5 interventions in health and medicine that improve clinical care are also cost-saving compared with usual care.

Moreover, although we have not modelled for indirect cost, i.e. the impact of the intervention on patients’ ability to work, there is increasing evidence that this cost category is reduced by way of prevented disease progression as well. Furthermore, if early accurate diagnosis leads to appropriate treatment, there should be significant downstream cost savings in medication and joint replacement surgeries [26, 27].

Overall, the assumptions made in the model are conservative, to minimize the risk of overstating the effects of the parameters within the model.

The purpose of our study is not to blame Austrian physicians in primary care for their lack of diagnostic skill - far from it. The authors are convinced that colleagues in primary care act as important and trustworthy counsellors who have known their patients - and very often patients’ immediate families - for years. All too often the burden of many chronic diseases rests on the shoulders of these physicians. Yet, according to our estimate, GPs see an average of 0.4 incident RA patients per year - not only is RA hard to diagnose, it is also rare.

Our results show that by better directing the flow of people with RA, simultaneous benefits may be reaped: clinical and economic benefits.

As treatment guidelines [6] increasingly take the economic impact of different therapeutic approaches into account, it is important to allocate resources so that health gain is maximised. Our analysis shows that rethinking the way patients have to go before receiving their correct diagnosis has the potential to free resources which can then be used to treat more patients or treat existing patients with innovative and, therefore, more costly therapeutics.

An important strength of our analysis certainly pertains to the local data on practice patterns, diagnostic accuracy and diagnostic measures we were able to use thanks to previous work published [9–11, 15]. We relied on first-hand information from both rheumatologists and primary care physicians.

As said above, the role of a GP is quite extensive; he is usually the first to see the patient with musculoskeletal disorders and has to decide whether or not to refer a patient to a rheumatologist. Moreover he has an important role in follow-up and surveillance. One of the main reasons for a delay in seeing a specialist are long waiting lists for rheumatologist appointments.

A limitation of our study is that the RAC approach, which is the pre-condition of our reconfiguration, is not yet available across the entire country. However, early diagnosis and rapid initiation are important for patients’ outcome and much more likely when early arthritis clinics and rapid assessment consultation are instituted. Of note, it has been recently shown with office based rheumatologists that overall workload and working hours before and after implementation of RAC were the same, however, with a different schedule due to regrouping of patients. A patient with a suspicion of an inflammatory rheumatic disease is seen within 1 week; patients in RAC without the need for specialist treatment can be identified quickly and are given appropriate recommendations for further care [11].

However, there are several other limitations the reader should be aware of when interpreting the study at hand. Firstly, we used a model, which per se is a depiction of the Austrian situation and not necessarily reality. While we think we are able to provide a fair estimate, our analysis relies on the model being accurate. While we modelled for the Austrian situation only and the local situation might not necessarily be applicable to health care systems in other countries, discussions with colleagues practicing in other European countries lead us to believe that the situations might be similar.

Another important limitation of our study is the epidemiological data our analysis is based on - there is, at this time, no nationwide registry. However, as this kind of data is very important to all aspects of health policy and priority setting in health care, we strongly advocate to support efforts geared at improving this situation. Moreover, this would greatly enhance transparency in health care decision-making.

## Conclusions

In conclusion, initiation of care by a rheumatologist early on in the disease offers the potential for dramatically improving clinical outcomes in patients with RA [13, 23]. Our work further underscores the importance of RAC, also in remote settings, as early diagnosis is crucial for patients’ prognosis. Lastly there is the potential of economic benefits.

## Abbreviations

ACPA: Antibodies against citrullinated protein/peptide antigens
ALT: Alanine transaminase gamma
CBC: Complete blood count
Crea: Creatinine
CRP: C-reactive protein
DMARD: Disease-modifying anti-rheumatic drug
ESR: Erythrocyte sedimentation rate
GGT: Glutamyltransferase
GP: General practitioner
HAQ: Health Assessment Questionnaire
IA: Inflammatory Arthritis
RA: Rheumatoid arthritis
RAC: Rapid Assessment Consultations
RF: Rheumatoid factor
UA: Undifferentiated arthritis
UA: Uric acid

## References

[1] Bykerk V, Emery P (2010). Delay in receiving rheumatology care leads to long term harm. Arthritis Rheum.

[2] Möttönen T, Hannonen P, Korpela M, Nissilä M, Kautiainen H, Ilonen J, FIN-RACo Trial Group (2002). Delay to institution of therapy and induction of remission using single-drug or combination-disease-modifying antirheumatic drug therapy in early rheumatoid arthritis. Arthritis Rheum.

[3] Nell VP, Machold KP, Eberl G, Stamm TA, Uffmann M, Smolen JS. Benefit of very early referral and very early therapy with disease-modifying, anti-rheumatic drugs in patients with early rheumatoid arthritis. Rheumatology (Oxford). 2004;43:906–14. 10.1093/rheumatology/keh199 15113999

[4] van der Linden MP, le Cessie S, Raza K, van der Woude D, Knevel R, Huizinga TW, van der Helm-van Mil AH (2010). Long-term impact of delay in assessment of patients with early arthritis. Arthritis Rheum.

[5] van Nies JA, de Jong Z, van der Helm-van Mil AH, Knevel R, Le Cessie S, Huizinga TW (2010). Improved treatment strategies reduce the increased mortality risk in early RA patients. Rheumatology (Oxford).

[6] Smolen JS, Landewé R, Breedveld FC, Buch M, Burmester G, Dougados M (2014). EULAR recommendations for the management of rheumatoid arthritis with synthetic and biological disease-modifying antirheumatic drugs. Ann Rheum Dis.

[7] Krüger K, Karberg K (2011). Treat-to-target from the perspective of office-based rheumatology. Z Rheumatol.

[8] Westhoff G, Edelmann E, Kekow J, Zink A (2010). Diagnostic spectrum, treatment indication and symptom duration in initial referrals to the rheumatologist. Z Rheumatol.

[9] Puchner R, Edlinger M, Mur E, Eberl G, Herold M, Kufner P, Puchner A, Puchner SE, Redlich K, Alkin A, Machold K (2016). Interface management between general practitioners and rheumatologists-results of a survey defining a concept for future joint recommendations. PLoS One.

[10] Gärtner M, Fabrizii JP, Koban E, Holbik M, Machold LP, Smolen JS (2012). Immediate access rheumatology clinic: efficiency and outcomes. Ann Rheum Dis.

[11] Puchner R, Kaiser W, Janetschko R, Linkesch A, Steininger M, Tremetsberger R, Machold K (2016). Efficacy and outcome of rapid access rheumatology consultation: an office-based pilot cohort study. J Rheumatol.

[12] Radner H, Smolen JS, Aletaha D (2014). Remission in rheumatoid arthritis: benefit over low disease activity in patient reported outcomes and costs. Arthritis Res Ther.

[13] Kyburz D, Gabay C, Michel BA, Finckh A, physicians of SCQM-RA. The long-term impact of early treatment of rheumatoid arthritis on radiographic progression: a population-based cohort study. Rheumatology (Oxford). 2011;50(6):1106–10. 10.1093/rheumatology/keq424 21258051

[14] Xu D, Groom C, Taylor M. Economic models of identification and treatment of early rheumatoid arthritis. National Audit Office. http://www.nao.org.uk/wp-content/uploads/2009/07/0809823_Economic_Model.pdf.

[15] Puchner R, Brezinschek HP, Herold M, Nothnagl T, Studnicka- Benke A, Fritz J, Leeb BF (2014). Quality of care of rural rheumatoid arthritis patients in Austria. Wien Klin Wochenschr.

[16] Tobon GJ, Youinou P, Saraux A (2010). The environment, geo-epidemiology, and autoimmune disease: rheumatoid arthritis. J Autoimmun.

[17] Symmons DPM (2005). Looking back: rheumatoid arthritis- aetiology, occurrence and mortality. Rheumatology.

[18] Wiles N, Symmons DP, Harrison B, Barrett E, Barrett JH, Scott DG, Silman AJ (1999). Estimating the incidence of rheumatoid arthritis: trying to hit a moving target?. Arthritis Rheum.

[19] Core Team R (2014). R: A language and environment for statistical computing.

[20] Gormley GJ, Steele WK, Gilliland A, Leggett P, Wright GD, Bell AL (2003). Can diagnostic triage by general practitioners or rheumatology nurses improve the positive predictive value of referrals to early arthritis clinics?. Rheumatology (Oxford).

[21] Walter E, Zehetmayr S (2006). Guidelines for health-economic evaluations in Austria. Wien Med Wochenschr.

[22] Puolakka K, Kautiainen H, Möttönen T, Hannonen P, Korpela M, Julkunen H (2004). Impact of initial aggressive drug treatment with a combination of disease-modifying antirheumatic drugs on the development of work disability in early rheumatoid arthritis: a five-year randomized followup trial. Arthritis Rheum.

[23] Finckh A, Liang MH, van Herckenrode CM, de Pablo P (2006). Long-term impact of early treatment on radiographic progression in rheumatoid arthritis: a meta-analysis. Arthritis Rheum.

[24] Huscher D, Merkesdal S, Thiele K, Zeidler H, Schneider M, Zink A (2006). Cost of illness in rheumatoid arthritis, ankylosing spondylitis, psoriatic arthritis and systemic lupus erythematosus in Germany. Ann Rheum Dis.

[25] Cohen JT, Neumann PJ, Weinstein MC (2008). Does preventive care save money? Health economics and the presidential candidates. N Engl J Med.

[26] Huscher D, Mittendorf T, von Hinüber U, Kötter I, Hoese G, Pfäfflin A, for the German Collaborative Arthritis Centres (2015). Evolution of cost structures in rheumatoid arthritis over the past decade. Ann Rheum Dis.

[27] Ziegler S, Huscher D, Karberg K, Krause A, Wassenberg S, Zink A (2010). Trends in treatment and outcomes of rheumatoid arthritis in Germany 1997-2007: results from the National Database of the German Collaborative Arthritis Centres. Ann Rheum Dis.

